# Health and economic impacts of introducing specific excise tax to waterpipe tobacco in Egypt: a simulation model of simple and mixed tax policy approaches

**DOI:** 10.1136/bmjgh-2023-012048

**Published:** 2023-10-09

**Authors:** Aya Mostafa, Ali Chalak, Rima Nakkash, Ruba Abla, Yousef S Khader, Niveen ME Abu-Rmeileh, Ramzi G Salloum, Mohammed Jawad

**Affiliations:** 1 Department of Community, Environmental and Occupational Medicine, Ain Shams University Faculty of Medicine, Cairo, Egypt; 2 Department of Agriculture, Faculty of Agricultural and Food Sciences, American University of Beirut, Beirut, Lebanon; 3 Global and Community Health Department, George Mason University, Fairfax, Virginia, USA; 4 Department of Health Promotion and Community Health, Faculty of Health Sciences, American University of Beirut, Beirut, Lebanon; 5 Department of Community Medicine, Jordan University of Science and Technology, Irbid, Jordan; 6 Institute of Community and Public Health, Birzeit University, Birzeit, State of Palestine; 7 Health Outcomes and Biomedical Informatics, University of Florida College of Medicine, Gainesville, Florida, USA; 8 Public Health Policy Evaluation Unit, Imperial College London School of Public Health, London, UK

**Keywords:** health policy, public health

## Abstract

**Introduction:**

Waterpipe tobacco is taxed at half the rate of cigarettes in Egypt and, unlike cigarettes, does not have a specific excise component. We aimed to simulate the introduction of a specific excise tax on waterpipe tobacco consumption, premature deaths and government waterpipe tobacco revenue in Egypt.

**Methods:**

We took model inputs from the latest available data on consumption, market shares and market share prices, price elasticities of demand, tax structure and from discussions with government officials. We modelled increases to specific excise to produce a 45%, 55%, 65% and 75% tax burden and compared a simple (specific only) structure with a mixed (specific and ad valorem) structure.

**Results:**

Under the simple approach, introducing a US$2.1 specific tax would result in a 75% tax burden with 67% fewer waterpipe tobacco units smoked, 1 004 604 averted premature deaths and a 236% increase in government revenue relative to the current tax structure. At the 75% tax burden, the simple approach resulted in 1.5% fewer waterpipe tobacco units consumed, 9000 more averted premature deaths and 12.7% more government revenue compared with the mixed approach. Results for other tax burdens are presented and remained robust to sensitivity analyses.

**Conclusions:**

Introducing a specific excise tax on waterpipe tobacco in Egypt can yield considerable government revenue and public health gains. We recommend the simple approach, in line with the WHO recommendations, which produces greater economic and public health gains than the mixed approach and is easier to administer for the Egyptian government.

**WHAT IS ALREADY KNOWN ON THIS TOPIC**

- In Egypt, waterpipe tobacco tax structure lacks a specific excise component, unlike cigarettes, and is unequally taxed at half its rate.
- Evidence supporting improvements in waterpipe tobacco tax structure in Egypt is lacking.

**WHAT THIS STUDY ADDS**

- This study models simple (specific only) and mixed (specific and ad valorem) tax policy approaches at five tax burdens.
- Introducing specific excise to waterpipe tobacco tax results in substantial fiscal and public health gains; we recommend a simple tax policy to a mixed tax policy.

**HOW THIS STUDY MIGHT AFFECT RESEARCH, PRACTICE OR POLICY**

- We provide policy makers the contextualised evidence for different tax policy approaches that could be framed as phased implementation through gradual and regular increase in tax burden.
- This model unfolds potential to improve government financing while protecting public health.

## Introduction

Tobacco taxation is a critical policy tool to effectively reduce tobacco use.^1^ It also generates a reliable source of government revenue to invest in advancing public health and development.^1^ However, the potential economic and public health benefits cannot be fully materialised if the tobacco tax policy is not at best-practice tax structure and level.^1^ Article 6 of the WHO Framework Convention on Tobacco Control (WHO FCTC) recommends including a specific excise component within a simple and uniform tax structure as a superior approach to ad valorem tax.^2^ Also, WHO recommends applying tax at 75% level or higher of the tobacco product retail price (the tax burden).^1^ To support improvements in tobacco tax policies, locally generated evidence is crucial to contextualise and optimise its political framing.^3^

This is of particular importance in the few countries where tobacco use is still rising,^4^ such as Egypt. The tobacco industry is expanding its production in Egypt as one of the largest cigarette and waterpipe tobacco markets in the world.^5^ In 2021, Egypt scored 64/100 on the tobacco industry interference index and ranks 52/80 countries; the government owns half of the local tobacco company that dominates the market.^5^ Tobacco taxes constitute approximately 8% of the national tax revenue; economic benefits derived from earmarked tobacco taxes are reinvested in financing the newly launched universal health coverage programme.^6^

Since 2010, Egypt’s government has been regularly increasing cigarette taxes,^7^ currently at a 78.5% tax burden,^1^ and has added a specific excise tax to ensure minimum revenue levels and greater reductions in consumption.^1^ However, waterpipe tobacco in Egypt has not received equally strong taxation measures and continues to have an ad valorem excise tax only.^8^ The total tax burden of waterpipe tobacco is reported as 70.9% (68.5% ad valorem and 2.4% import duty) by WHO based on the price of one brand and is not market-weighted^8^; our research group believes this value would be less inflated if market shares and prices are taken into account, providing a more realistic picture of the Egyptian waterpipe tobacco policy. In 2020, the price of 20 g of waterpipe tobacco (US$0.47) was less than half of a pack of cigarettes (US$1.07).^8^ This non-uniform taxation may lead to shifting to cheaper types of tobacco products,^2^ especially amid the global price increases.

The relative waterpipe tobacco affordability in Egypt is worrisome because of the soaring youth smoking rates. Adolescent girls report higher smoking prevalence rates than adult women regarding both waterpipe tobacco (4.1% vs 0.1%) and cigarettes (0.8% vs 0.3%).^9 10^ In adolescent boys, smoking prevalence rates are still lower than those reported in adult men, however, the gap is much closer for waterpipe tobacco (7.2% vs 8.7%) than cigarettes (8.3% vs 35.9%). Moreover, waterpipe tobacco and cigarette smoking rates reach up to a quarter among high school students (21% and 28%, respectively)^11^ and university students (21.6% and 28.3%, respectively).^12^ Most adult waterpipe tobacco smokers (66.7%) smoke it daily in cafes and at home (51.6%), exposing other household members to second-hand smoke,^10^ and are unaware of the associated disease risk (including cancer, respiratory and cardiovascular diseases),^13^ toxicant inhalation and dependence risk that are similar to cigarettes.^14^ A quarter of Egyptian smokers reported waterpipe tobacco dependence.^15^ Furthermore, tobacco costs Egypt 90 billion Egyptian Pound (EGP) (2.1% of the GDP) annually, of which 75% is due to premature mortality.^16^

Consistent evidence regarding cigarette taxation positive impacts on smoking cessation, public health outcomes and government revenue was generated through tobacco tax simulation models, such as SimSmoke, the WHO Tobacco Tax Simulation Model and the Tobacco Excise Tax Simulation Model (TETSiM).^17^ To date, similar evidence for waterpipe tobacco is greatly lacking. Our research group used TETSiM to publish the first study examining waterpipe tobacco taxation impacts in Jordan, Lebanon and Palestine.^18^ Waterpipe tobacco use is prevalent in many of the Eastern Mediterranean countries, yet waterpipe tobacco tax levels are still lower than the global average (48.9%) and local cigarette tax rates.^8 18^ We found that increasing waterpipe tobacco-specific excise taxes substantially reduced smoking and premature deaths, and increased government revenues in these countries.^18^

Reforming the waterpipe tobacco tax structure will support Egypt in achieving both global and national public health targets: the Sustainable Development Goal target 3a and Egypt’s vision 2030 first target.^19 20^ To inform a more effective waterpipe tobacco taxation policy, we simulated the introduction of a specific excise tax component to Egypt’s waterpipe tobacco tax structure through different tax policy approaches, and modelled their impact on waterpipe tobacco consumption, averted premature deaths and government revenue.

## Methods

### Overview

We developed a cross-sectional, arithmetic model adapted from TETSiM^21^ that estimated the quantitative impact of an excise tax change on waterpipe tobacco consumption, government tax revenue and premature deaths averted in Egypt. We took model inputs from the latest available waterpipe tobacco data (consumption, market shares and market share prices, price elasticities of demand and tax structure), where available, and from discussions with the Egyptian Tax Authority.^22^ We modelled various excise tax changes that increased the current waterpipe tobacco tax burden (total tax as percentage of the retail price) in Egypt under two tax policy approaches: ‘simple’ (specific excise only) and ‘mixed’ (specific and ad valorem excise).

### Model data inputs

All model data inputs are presented in table 1.

**Table 1:** Waterpipe tobacco taxation main model data inputs for Egypt

Variable	Input value	Date (source)
Waterpipe tobacco consumption		
Current (daily and non-daily use) waterpipe tobacco prevalence, %	4.5	2017^10^
Current daily use, %	3.0	2017^10^
Number of 20 g units smoked by daily smokers in their last session, mean	2.8	2017^10^
Number of sessions smoked by daily smokers per day, mean	3.6	2017^10^
Population size aged >15 years	67 589 102	2020^40^
Number of annual 20 g sessions	6 672 334 214	2017, 2020^10 40^
Illicit (non-tax paid) consumption, % of current use	3.5	2021^24^
Market shares and market share prices		
Café flavoured share, %	18.9	2015–2017^15 25–28^
Café unflavoured share, %	27.7	2015–2017^15 25–28^
Home flavoured share, %	2.1	2015–2017^15 25–28^
Home unflavoured share, %	51.3	2015–2017^15 25–28^
Café flavoured expenditure per 20 g, US$	2.09	2015–2017^15 25–28^
Café unflavoured expenditure per 20 g, US$	0.20	2015–2017^15 25–28^
Home flavoured expenditure per 20 g, US$	2.11	2015–2017^15 25–28^
Home unflavoured expenditure per 20 g, US$	0.07	2015–2017^15 25–28^
Price elasticity of demand		
Café flavoured own-price elasticity of demand	0.489	2000, 2015–2017, 2019^15 25–28 31 32^
Café unflavoured own-price elasticity of demand	0.445	
Home flavoured own-price elasticity of demand	0.321	
Home unflavoured own-price elasticity of demand	0.592	
Tax structure		
Specific excise tax per 20 g, US$	0.0	2022^22^
Import duty, % of CIF/ex-factory price	0.0% (unflavoured) 16.7% (flavoured)	2022^22^
Ad valorem excise tax, % of CIF/ex-factory price and import duty	165% (unflavoured) 200% (flavoured)	2022^22^
Value added tax, % retail price	0.00	2022^22^
Annual imports (code 240311) for waterpipe tobacco, trade value, US$	1 151 028	2022^33^
Annual imports (code 240311) for waterpipe tobacco, netweight, kg	76 824	2022^33^
CIF/Ex-factory price per 20 g of café unflavoured waterpipe tobacco, US$	0.07	2022^22^
CIF/Ex-factory price per 20 g of home unflavoured waterpipe tobacco, US$	0.02	2022^22^
CIF/Ex-factory price per 20 g of café/home premium waterpipe tobacco, US$	0.30	2022^33^
Inflation and exchange rates		
Inflation rate, %	29.8	2017^29^
Inflation rate, %	14.4	2018^29^
Inflation rate, %	9.4	2019^29^
Inflation rate, %	5.6	2020^29^
Inflation rate, %	4.9	2021^29^
Inflation rate, %	13.2	2022^29^
Exchange rate, EGP to US$	0.0531892	Mid-year 2022^30^

CIF, cost, insurance and freight; EGP, Egyptian Pound.

#### Waterpipe tobacco consumption

In line with the 2021 WHO Report on the Global Tobacco Epidemic,^1^ we set the waterpipe tobacco unit at 20 g, which is the estimated weight used in one smoking session.^23^ We used the latest national data on current (daily and less than daily) waterpipe tobacco smoking prevalence (4.5%) reported from the 2017 STEPwise survey of non-communicable disease risk factors (STEPS) in individuals aged 15–69 years.^10^ This survey captured both legal and illicit consumption, so we downscaled our outcomes by 3.5% under the assumption that waterpipe illicit rates were the same as for cigarettes.^24^

#### Market shares and market share prices

We took market share and price data from a recently published cross-sectional survey that was conducted between 2015 and 2017 among 1490 adult (aged ≥18 years) waterpipe tobacco smokers living in Cairo and a rural area in the Nile Delta.^15 25–28^ The survey captured the self-reported usual waterpipe tobacco smoking location, the average number of waterpipe tobacco 20 g units smoked per day and the average daily waterpipe tobacco expenditure (in EGP).^15 25–28^

Waterpipe tobacco prices vary substantially depending on the presence of flavour and location of use,^18^ which we reflected in four market shares: (1) flavoured waterpipe tobacco in a café (18.9% share, US$2.09 mean expenditure per 20 g), (2) unflavoured waterpipe tobacco in a café (27.7%, US$0.17), (3) flavoured waterpipe tobacco at home (2.1%, US$2.11) and (4) unflavoured waterpipe tobacco at home (51.3%, US$0.07). Prices were inflation-adjusted to 2022^29^ and we used an exchange rate of 0.053 EGP/US$ in mid-year 2022.^30^

#### Price elasticity of demand

To the best of our knowledge, the only estimate for the price elasticity of demand in Egypt was made in the year 2000 for cigarettes (−0.397)^31^ and no studies have estimated waterpipe tobacco price elasticities in Egypt. Therefore, we inferred data from recently published price elasticity estimates conducted in Jordan, Lebanon and Palestine.^32^ Based on expert advice, we calculated Egypt’s waterpipe tobacco price elasticity estimates for each market share in three steps. Step 1: we calculated the ratio between waterpipe tobacco and cigarette own-price elasticity estimates for each market share in each country (online supplemental table S1).^32^ Step 2: we calculated the average of each market share’s ratio. Step 3: we applied the average of each market share’s ratio to Egypt’s cigarette price elasticity estimate, to obtain Egypt’s waterpipe tobacco price elasticity estimate for each market share:. café/flavoured −0.489; home/flavoured −0.321; café/unflavoured −0.445; home/unflavoured −0.592 (online supplemental table S1). We believe this approach was more valid than simply using cigarette price elasticities (given vastly different consumer behaviour between the two products), or taking the arithmetic mean of waterpipe elasticities from neighbouring countries (given their wide statistical dispersion) or using elasticities from one of the three countries (given the social differences in which waterpipe tobacco is smoked).

10.1136/bmjgh-2023-012048.supp1 Supplementary data

#### Tax structure

We used the waterpipe tobacco tax structure available in Law no. 13 for 2020 and confirmed the method of its administration in 2022 during discussions with the Egyptian Tax Authority.^22^ We assumed that all flavoured waterpipe tobacco products were imported as premium, and that all unflavoured products were domestically produced. For flavoured products, we obtained import prices (also known as costs, insurance and freight (CIF) prices) from the United Nations Comtrade depository for international trade data (product code 240311), where import weights and prices were reported in US$.^33^ Based on the latest data year available for Egypt (2021), we calculated the CIF value by dividing the annual imports trade value in US$ by the annual imports net weight quantity in 20 g waterpipe tobacco units,^33^ then adjusting for inflation in 2022.^30^ Following discussions with the Egyptian Tax Authority,^22^ (a) we estimated ex-factory prices for domestically produced and unflavoured waterpipe tobacco as US$0.02 for discount home use and US$0.07 for discount café use, (b) we set the ad valorem excise tax at 165% for local products and 200% for imported products, and the import tax at 2.4% and (c) we confirmed that import duty was applied to the CIF price, that the specific excise tax was applied to each 20 g unit of waterpipe tobacco and that the ad valorem excise tax was applied to the sum of CIF/ex-factory and import duty (value-added tax is not applied). The industry margin was calculated as the retail price minus the sum of all taxes and the CIF/ex-factory price. The ad valorem excise tax rates (165%–200%) differ from the tax burden (eg, 75%) as the denominator of the former is the sum of the CIF/ex-factory price and import duty while the denominator of the latter is the total retail price.

### Model structure

Egypt uses only an ad valorem excise tax on waterpipe tobacco products. We decomposed the retail price of 20 g of waterpipe tobacco into five categories unique to each market share: the CIF/ex-factory price, import duty, specific excise tax, ad valorem excise tax and the industry margin. For each market share, we calculated the annual number of waterpipe tobacco 20 g units as a product of the mean number of waterpipe tobacco sessions smoked by daily users in their last waterpipe tobacco session (2.8),^10^ the mean number of waterpipe tobacco sessions smoked per day (3.6),^10^ the prevalence of daily use (3.0%)^10^ and the population size from the World Bank corresponding to the age range in STEPS (60 409 575)^34^ by 365.25 days (table 1). The product of the annual number of waterpipe tobacco 20 g units and excise duties produced the annual government revenue from excise for each market share. We calibrated the base scenario model to ensure annual government revenue matched official statements that estimated the revenue from waterpipe tobacco was 30% (US$1.1 billion) of all tobacco tax revenues (US$3.8 billion) in 2022.^22^

To estimate potential consumption values following a change in underlying excise tax rates from baseline (period 0) to follow-up (period 1), we used the following formula:

$$Q_{1m}=Q_{0m}{\left(P_{1m}/P_{0m}\right)}^{\varepsilon_{m}}$$

where *Q* is the quantity of 20 g units of waterpipe tobacco consumed, *P* is the retail price, *m* is the market share and *ε* is the own-price price elasticity of demand. This formula avoids overestimating demand responses where tax increases are large, by basing our simulations on an algebraically ‘exact’ (ie, exponential decay) rather than ‘approximate’ (ie, linear decrease) relationship between per cent price and demand changes that is captured by price elasticities.^18^ New consumption values were multiplied by new excise rates to calculate the new annual government revenue from excise duties.

We estimated premature deaths averted based on an assumption derived from cigarette modelling that 50% of the reduction in waterpipe tobacco sessions smoked was due to smokers quitting (rather than reducing their consumption), of whom 35% may have died prematurely had they not quit.^21 34^ We believe this assumption could be applied to waterpipe tobacco; a meta-analysis reported that harms resulting from using cigarettes and waterpipe tobacco are similar.^13^

### Analysis

The base scenario reflected current waterpipe tobacco consumption and government revenue from waterpipe tobacco taxes in Egypt in 2022.^10 22^ By removing the ad valorem tax and only varying the specific tax ‘simple approach’ (for a uniform simple tax structure, as recommended by the WHO FCTC^2^), we modelled five scenarios that resulted in 45%, 55%, 65% and 75% tax burdens, in addition to a 48.9% tax burden which allowed us to compare with the global average tax burden on waterpipe tobacco in 2021.^8^ For comparative purposes, a secondary analysis ran these models by varying the specific tax without removing ad valorem rates ‘mixed approach’. For each scenario, our three outcomes were the annual number of 20 g units consumed, the annual number of premature deaths averted and the annual total government revenue from waterpipe tobacco taxation (sum of import, specific and ad valorem taxes).

In modelling different tax scenarios, we assumed no changes in the illicit trade rate, and we assumed production costs remained constant and the industry passed the whole cost of the tax onto the consumer without overshifting or undershifting their prices in response, that is, no changes in the CIF/ex-factory price or industry margin.^35^

In a secondary analysis, we changed our model estimates to match the WHO tax structure data,^1 1^ which states that the tax burden on 20 g of waterpipe tobacco in Egypt is already 70.4%.^8^ For this model, we used one market share on which the data were based following discussion with WHO (premium home) which had a CIF of US$0.09, import duty ofUS$0.01, ad valorem of US$0.32, industry margin of US$0.05 and retail price of US$0.47. As per our specific approach, our secondary model removed the ad valorem component and included a specific excise duty that ensured the tax burden reached 75%.

The TETSiM model does not capture risk factor data for premature mortality beyond tobacco use and does not project into time; we did, however, amalgamate a year’s worth of waterpipe tobacco sessions in Egypt but analysed it as a cross-sectional dataset.

### Sensitivity analyses

To address underlying possible uncertainties or potentially highly influential parameters, we performed sensitivity analysis for three assumptions. Our first assumption was related to Egypt’s waterpipe tobacco price elasticity estimate in each of the four market share categories, so we altered these to the lower and upper 95% CIs of the original estimate. The second uncertainty varied the CIF/ex-factory price by 50% below or above the original CIF price. The third uncertainty was the assumption about the industry non-response to a tax change, so we included a 10% industry overshift or undershift. The impact of the sensitivity analyses results on waterpipe tobacco consumption, premature deaths averted and government tax revenue are reported at the 75% scenario only, expressed as a relative per cent difference from the original 75% scenario analysis.

### Public involvement

During the preparatory stage for this research, members of the public were involved in a policy dialogue to deliberate on restructuring the waterpipe tobacco taxation policy in Egypt. The priority of the research question, the inclusion of all relevant evidence, the overall framing and contextualisation of the problem of waterpipe tobacco smoking in Egypt and the possible tax policy approaches were informed by discussions with 17 diverse stakeholders, including the civil society. The publication will be shared through www.egypttobaccoobservatory.com. Also, a study newsletter suitable for a non-specialist audience will be shared. This study is an economic model, and no patients were recruited or involved.

## Results

Tax structures and market-weighted retail prices of 20 g of waterpipe tobacco for each simulated scenario under both (simple and mixed) approaches are presented in table 2. Based on our data for the specific prices for the four market shares, quantities consumed and tax structure, we calculated that only 39.1% of the market-weighted retail price was tax (across all waterpipe tobacco market shares). In the base scenario where no specific excise taxes were added, the weighted average retail price of 20 g of waterpipe tobacco was US$0.53 (table 2), and adults in Egypt consumed 3.7 billion 20 g units of waterpipe tobacco annually and the government revenue was US$763 million (table 3).

**Table 2:** Tax structures and market-weighted retail prices of 20 g of waterpipe tobacco for each simulated scenario under both approaches (simple and mixed), US$

Scenario	CIF price	Import duty	Specific excise	Ad valorem excise	Retail price
Approach 1: simple specific excise tax structure					
Base scenario, 39.1% tax burden	0.09	0.01	0.00	0.00	0.53
45% tax burden	0.14	0.02	0.44	0.00	1.02
48.9% tax burden	0.15	0.02	0.54	0.00	1.14
55% tax burden	0.16	0.02	0.73	0.00	1.37
65% tax burden	0.17	0.02	1.20	0.00	1.89
75% tax burden	0.17	0.03	2.08	0.00	2.81
Approach 2: mixed specific and ad valorem excise tax structure					
Base scenario, 39.1% tax burden	0.09	0.01	0.00	0.02	0.53
45% tax burden	0.11	0.01	0.08	0.24	0.72
48.9% tax burden	0.12	0.01	0.15	0.26	0.86
55% tax burden	0.13	0.02	0.31	0.29	1.12
65% tax burden	0.15	0.02	0.74	0.33	1.68
75% tax burden	0.16	0.02	1.58	0.37	2.63

CIF, costs, insurance and freight.

**Table 3:** The impact of different waterpipe tobacco taxation scenarios on waterpipe tobacco consumption, premature deaths averted, government revenue and retail price, and the relative per cent change from the base case under both (simple and mixed) approaches in Egypt

Scenario	Quantity consumed (20 g units) N (%)*	Premature deaths averted N	Government tax revenue (US$) N (%)*	Retail price, US$ (%)
Approach 1: simple specific excise tax structure				
Base scenario, 39.1% tax burden	3 670 117 434	–	763 181 540	0.53
45% tax burden	2 098 132 611 (−42.8)	486 337	966 435 232 (+26.2)	1.02 (+92.4)
48.9% tax burden	1 966 527 727 (−46.4)	552 498	1 098 160 190 (+43.9)	1.14 (+114.7)
55% tax burden	1 775 745 364 (−51.6)	654 278	1 334 703 216 (+74.9)	1.37 (+156.9)
65% tax burden	1 490 454 880 (−59.4)	821 861	1 829 431 515 (139.7)	1.89 (+255.0)
75% tax burden	1 214 063 015 (−66.9)	1 004 604	2 560 543 454 (+235.5)	2.81 (+428.6)
Approach 2: mixed specific and *a*d valorem excise tax structure				
Base scenario, 39.1% tax burden	3 670 117 434	–	763 181 540	0.53
45% tax burden	2 870 337 355 (−21.8)	270 252	931 139 097 (+22.0)	0.72 (+35.5)
48.9% tax burden	2 511 841 110 (−31.6)	403 189	1 058 673 356 (+38.7)	0.86 (+62.0)
55% tax burden	2 101 652 136 (−42.7)	569 750	1 288 197 118 (+68.8)	1.12 (+109.5)
65% tax burden	1 630 923 904 (−55.6)	791 071	1 778 350 300 (+133.0)	1.68 (+215.4)
75% tax burden	1 270 673 487 (−65.4)	995 521	2 509 369 732 (+222.8)	2.63 (+395.0)

*Relative per cent change from base scenario.

**Figure 1:**
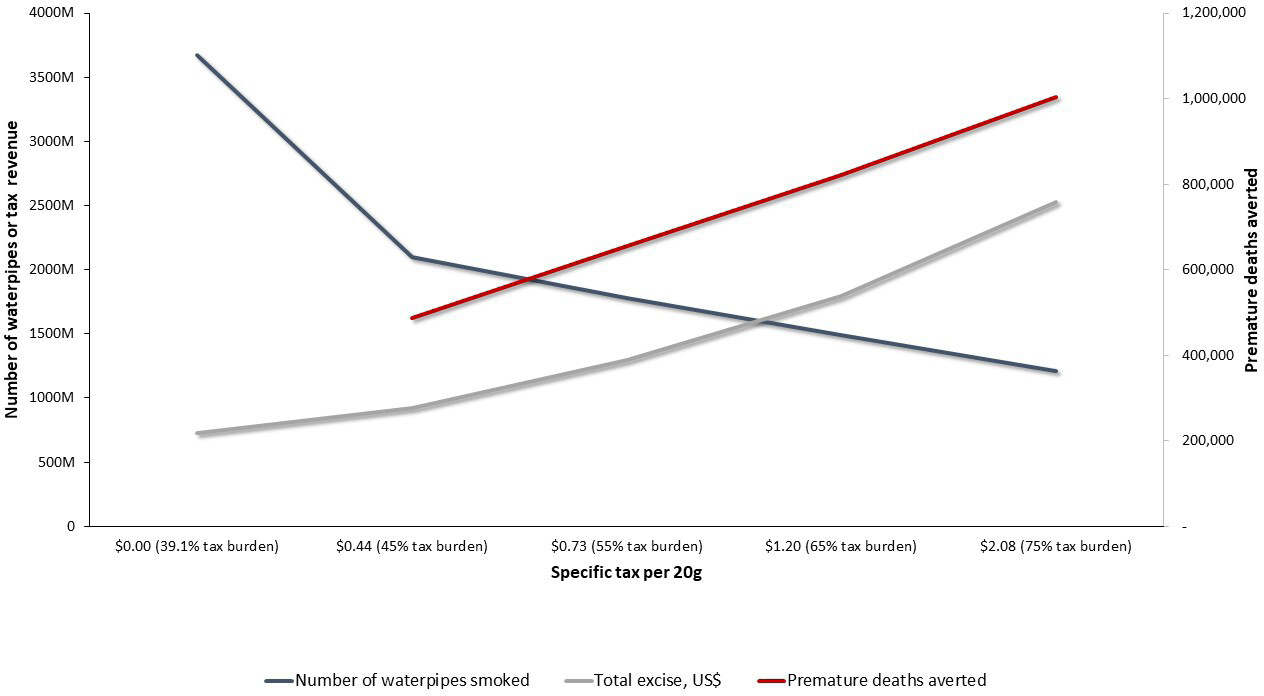
The impact of waterpipe tobacco tax increases on the annual number of waterpipe tobacco units smoked (blue line), premature deaths averted (red line) and government revenue from excise tax (grey line), by the amount of specific excise tax per waterpipe tobacco unit (20 g) and market-weighted tax burden under the simple’ (specific excise tax only) approach in Egypt.

**Figure 2:**
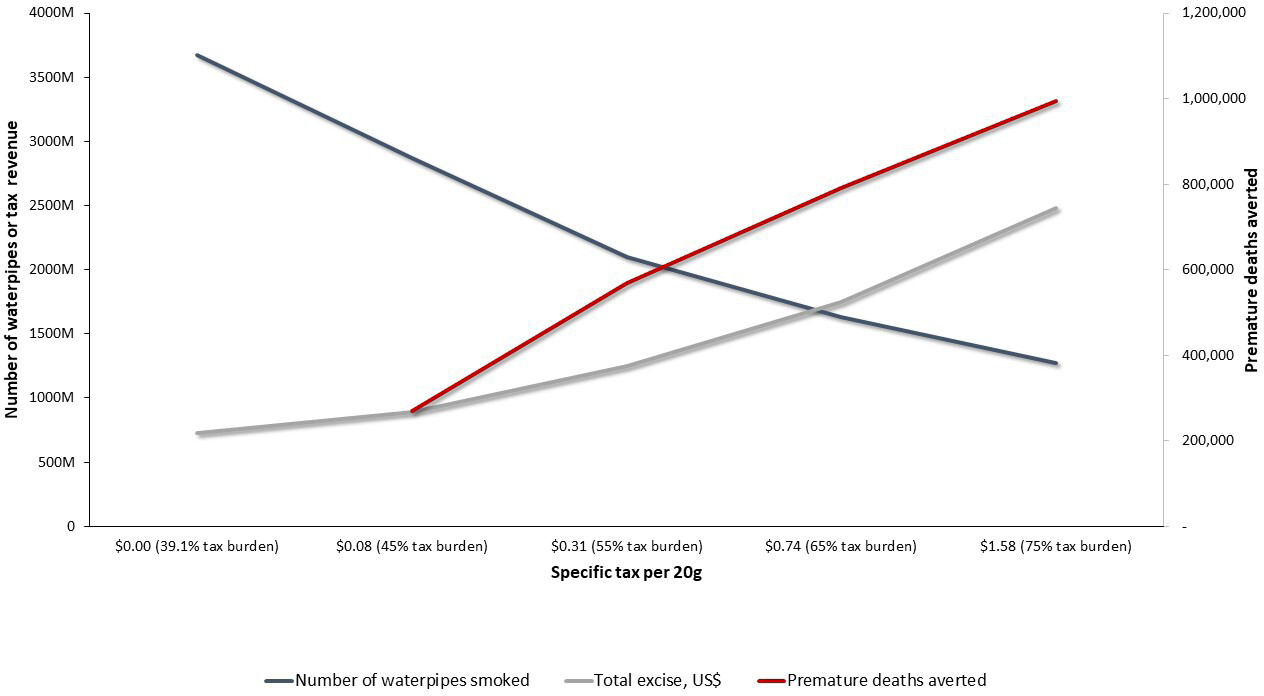
The impact of waterpipe tobacco tax increases on the annual number of waterpipe tobacco units smoked (blue line), premature deaths averted (red line) and government revenue from excise tax (grey line), by the amount of specific excise tax per waterpipe tobacco unit (20 g) and market-weighted tax burden under the mixed’ (specific and ad valorem excise tax) approach in Egypt.

Table 3 summarises the impacts of our modelling simulations for different waterpipe tobacco taxation scenarios on waterpipe tobacco consumption, premature deaths averted and government revenue under both (simple and mixed) approaches. They are graphically summarised in figures 1 and 2. Under the simple approach, if the specific excise tax is increased to US$0.73 per 20 g of waterpipe tobacco in the 55% scenario, Egypt could reduce waterpipe tobacco consumption by 52%, avert 654 000 premature deaths and increase government revenue by 75% (table 3). In this scenario, the weighted average retail price of 20 g of waterpipe tobacco is 2.6 times that in the base scenario (US$1.37 vs US$0.53) (table 2). In the same scenario under the mixed approach, the required specific excise tax is almost half that required for the simple approach (US$0.86 vs US$1.37). However, the simple approach will result in greater reductions in waterpipe tobacco consumption (9%), more averted premature deaths (85 000) and higher government revenue (6%) than the mixed approach at the 55% waterpipe tobacco tax rate (tables 2 and 3).

Under the simple approach, the 75% scenario would result in 67% fewer waterpipe tobacco 20 g units smoked, 1 004 604 averted premature deaths and a 236% increase in government revenue relative to the base scenario. In the 75% scenario, Egypt would need to increase the specific excise tax to S$2.08, which will increase the weighted average retail price of 20 g of waterpipe tobacco to US$2.18, that is, 4 times that in the base scenario (tables 2 and 3). In the same scenario under the mixed approach, the required specific excise tax is three-quarters that required for the simple approach (US$1.58 vs US$2.08). However, the simple approach will also result in greater benefits (1.5% greater reductions in waterpipe tobacco consumption, 9000 more averted premature deaths and 12.7% higher government revenue (6%))—that are more tangible in economic rather than public health benefits when compared with the 55% scenario (tables 2 and 3).

In the scenario meeting the global average for waterpipe tobacco tax rate (48.9%), the simple approach will result in 14.8% greater reduction in waterpipe tobacco consumption, 149 000 more averted deaths and 5% higher government revenue—than the mixed approach (tables 2 and 3). The specific excise tax required to meet this tax rate in the simple approach is 5 times higher than that required for the mixed approach (US$0.73 vs US$0.15). In this scenario under the simple approach, the weighted average retail price of 20 g of waterpipe tobacco will be US$1.14, that is, 2.2 times that in the base scenario.

In online supplemental table S2, the largest reduction in the waterpipe tobacco consumption at the 75% scenario from the base case under both (simple and mixed) approaches (84.3%–86%) will be in the home unflavoured waterpipe tobacco market share, and it will be replaced by café flavoured use as the largest market share.

In our secondary analysis, which only included one market share to match the WHO tax structure data, we found that removing the ad valorem excise and adding a US$0.40 specific excise would result in a 75% tax burden and increase the retail price from US$0.47 to US$0.55 per session. This scenario would result in 5% fewer waterpipe tobacco 20 g units smoked, 25 090 averted premature deaths and a 17% increase in government revenue relative to the base scenario.

### Sensitivity analyses

In terms of direction and magnitude, results for effect estimates in the 75% scenario remained robust to sensitivity analyses (online supplemental table S3). There was a small effect on model outcomes (by 0.8%–2.0%) when we changed the CIF value (50% increase or decrease) and a small effect on model outcomes (by 0.7%–1.1% depending on the outcome) when we introduced a 10% change in the industry overshift or undershift (online supplemental table S3). There were larger effects on outcomes when we changed elasticity estimates (by lower and upper 95% CIs of the original value). This was most apparent for unflavoured waterpipe tobacco elasticities because it had largest market shares at either café (27.7%) or home (51.3%) use. For instance, change in elasticity estimates for unflavoured waterpipe tobacco market share resulted in 1.5%–18.0% change in outcomes for home and 5.7%–41.9% for café use. As a result of the increase or decrease in elasticities of the four market shares for three outcomes (ie, 24 sensitivity analyses), our outcomes changed by <5% in 11, by >5% and <15% in 8, and by >15% in five analyses (online supplemental table S3).

## Discussion

Our waterpipe tobacco model estimated that introducing a specific excise tax component to Egypt’s current waterpipe tobacco tax structure under both policy approaches, the simple (specific excise only) or mixed (specific and ad valorem excise), can yield considerable government revenue and public health gains. Moreover, the economic and public health gains under the simple approach are higher than those gained under the mixed approach in all of the five proposed tax scenarios. Compared with the current situation, waterpipe tobacco taxation scenarios that result in a 75% tax burden under the simple approach can more than double the government revenue, reduce waterpipe tobacco consumption by two-thirds and avert 1 004 604 premature deaths.

The amount of specific excise tax that is required for implementing this change at the 75% scenario under the simple approach is large (US$2.08) and the total retail price of a 20 g waterpipe tobacco unit would reach 4 times its current value. Although this high increase in retail price in the 75% scenario in Egypt would suppress waterpipe tobacco consumption to approximately a third of its current rate, it would be concerning for waterpipe tobacco producers and smokers who will both exert pressure on policymakers to dismiss such taxation policy. Therefore, a phased approach in applying gradual and regular increases in the specific excise tax burden was recommended during a policy dialogue with participation of high-level government officials representing the concerned ministries, members of parliament, civil society, health professionals, academic researchers and WHO to discuss a policy brief for restructuring waterpipe tobacco tax in Egypt. For instance, the government may start by implementing the 55% approach—that will include only a third of the specific excise tax amount that is required for the 75% scenario and will result in a much lower total retail price (US$1.14) than the 75% scenario—then apply further increases in 5% or 10% increments in a regular fashion (as done in cigarette tax). The 55% scenario could almost double the government revenue, cut down the consumption to one half and avert 654 000 premature deaths. We recommend starting with the 55% scenario rather than the 48.9% scenario that meets the global average of waterpipe tobacco tax because the latter can result in large reductions in consumption (only 5%–11% less than the 55% scenario under the simple and mixed approaches, respectively) without increasing government revenue gains as considerably (30%–31% less than the 55% scenario under the simple and mixed approaches, respectively). However, the high specific excise tax required to meet the 75% scenario in Egypt is still less than that required to meet a much lower waterpipe tobacco tax rate scenario (35.9%) in Lebanon (US$2.44), and to double the government revenue in Palestine (US$4.11).^18^ These findings highlight the role of locally generated evidence in contextualising and politically framing the waterpipe tobacco tax policy in each country.

Notably, a significant proportion of Egyptian waterpipe tobacco smokers (46.6%) use the product in cafes, where the retail price is on average 3.5 times more expensive per waterpipe tobacco 20 g unit than smoking at home, and where at least 15%–45% of retail price is industry margin. This large price disparity between café and home use results in highly inequitable tax impacts across market shares. Currently, taxes for flavoured market shares are at 35% of the total retail price for 20 g waterpipe tobacco. Thus, to achieve a policy objective where waterpipe tobacco tax constitutes a weighted market average of 75% of the retail price per 20 g waterpipe tobacco, a rather large specific excise tax component must be implemented to balance the uneven café-based industry profit. Such large increases in the specific tax may result in major suppression in home use demand. Indeed, our model showed that unflavoured waterpipe tobacco market shares were suppressed quickly because they are cheap at baseline. Our model also shows that cafés become the predominant market share in the 75% scenario, regardless of market share-specific price elasticities.

To our knowledge, this study is the first to simulate waterpipe tobacco taxation in Egypt. However, there are scarce simulations that examined the health and economic impacts of cigarette taxation in Egypt.^36 37^ In 2010, cigarette smoking prevalence was 16.3% in Egypt and cigarette taxes were 50% of retail price, and government revenue was US$1.1 billion. In that year, a cigarette tax simulation reported that raising tax to 70% of the retail price would reduce cigarette smoking by 12.5%, prevent >600 000 premature deaths and raise government tax revenues by US$939 million.^36^ In 2016, an abridged SimSmoke study reported that raising cigarette excise taxes from 72.5% to 75% of the retail price would reduce cigarette consumption by 1.9% in the first 5 years and by 3.8% in the longer term of policy implementation, and avert 129 300 premature deaths.^37^ However, cigarette smoking prevalence increased from 16.3% in 2010 to 18.6% in 2020,^4^ although cigarette tax burden in Egypt was 78.5% per the WHO estimates,^1^ and government revenues from smoked tobacco products (mainly cigarettes) in 2020/2021 were US$5 billion.^6^ Although government tax revenues increased due to increased cigarette taxation, cigarette consumption was not reduced as expected. This entails an in-depth investigation of the social, political and market forces that contributed to this phenomenon. The comprehensive implementation of combined tobacco control policies, such as smoke-free environments, cessation services, warnings against the dangers of tobacco use and raising tobacco taxes could more effectively produce the desired public health gains.^37^ In the case of waterpipe tobacco tax models, policy makers should keep in mind its unique smoking characteristics besides the different factors that influence its market share and prices, such as the larger industry margins at baseline that require more substantial tax increases. From a policy perspective, the mixed model would be the likely implementation approach by Egypt’s government given it is already used in their cigarette tax system. However, we propose adopting the simple approach in line with the WHO recommendations, being logistically more feasible and yielding higher economic and public health gains than those under the mixed approach in all five proposed tax scenarios.

### Strengths, challenges and limitations

The strengths of this study include the use of a peer-reviewed model structure that tests only changes in specific excise taxes. The model results could be easily comprehended by policy makers and were robust to sensitivity analyses. The inclusion of premature deaths averted as an outcome further supports the case for increased taxation on waterpipe tobacco products. Prices, quantities consumed, price elasticities and location of use were disaggregated by market share to provide a deeper understanding of the impact of different waterpipe tobacco taxation scenarios. We were able to demonstrate the limited value of reporting single market share tax and price data in the WHO Report of the Global Tobacco Epidemic, and its future iterations should include tax structures by market share to support a more accurate understanding of how tax changes will impact public health outcomes. We also show that the WHO data on waterpipe tobacco taxation structure, in its use of a single market share, underestimates the increase in specific tax required to meet a 75% tax burden (US$0.40%) compared with both our simple (US$2.08) and mixed (US$1.58) approaches that use a more detailed waterpipe tobacco structure with four market segments. Variables used in this model could be incorporated into routine tobacco surveys to inform waterpipe tobacco taxation policy decisions more effectively.

The main challenge in presenting accurate simulation results from this economic model was the lack of one survey source that contained updated national data on waterpipe tobacco smokers’ behaviour regarding consumption and expenditure. Although there are regular national surveys conducted by the government, such as the household income, consumption and expenditure survey by the Central Agency for Public Mobilization and Statistics,^38^ data collected do not contain information on the waterpipe tobacco quantity consumed. On the other hand, data in the 2017 STEPS Report^10^ do not contain information about waterpipe tobacco expenditure, despite reporting prices for cigarettes. Also, raw data from STEPS are owned by the Ministry of Health and Population and is not accessible. Therefore, we could only use data presented in the published STEPS Report^10^ and its associated STEPS databook provided by WHO. Accordingly, we resorted to two sources for presenting the latest available data on these model parameters: STEPS for national waterpipe tobacco smoking prevalence^10^ and the recently published survey for market shares and market share prices.^15 25–28^ The latter survey did not aim to measure waterpipe tobacco smoking prevalence, but it measured several aspects of waterpipe tobacco smoking behaviour,^15 25–28^ noting that the sample of current waterpipe tobacco smokers in the latter survey (n=1490) was 5 times larger^15 25–28^ than the sample in STEPS (4.5% current waterpipe tobacco smoking prevalence×6680 total sample in SETPS; ie, n~300 current waterpipe tobacco smokers only).^10^

Also, the lack of published waterpipe tobacco price elasticity estimates on the global and local levels did not enable us to use country-specific waterpipe tobacco price elasticities. The Ministry of Finance does not publicly provide information on waterpipe tobacco market shares, market share prices, price elasticities and waterpipe tobacco tax revenues. The only source of relevant information is news outlets, which are not always reliable and could be affected by tobacco industry interference.^17^ Moreover, conducting new national household surveys to obtain such information is expensive and impractical. Therefore, we had to adapt waterpipe tobacco price elasticities from three neighbouring countries that were produced by our research group^32^ as the best possible solution to bridge this knowledge gap; the alternative was to use a two-decade old local cigarette price elasticity estimate (−0.397)^31^ for all four market shares in the model. However, the waterpipe tobacco price elasticity estimates we calculated for Egypt were not too far from that local cigarette price elasticity figure (−0.321 to −0.592).

There are limitations related to the model design and assumptions as well. First, its design did not allow extended projections on consumption, government revenue and premature deaths averted. However, data inputs could be used in future dynamic simulation models. Second, the TETSiM model assumes price elasticities are constant in all scenarios but this would be unlikely in the scenarios with large retail price rises. Third, the government revenue estimates are likely to be conservative because indirect fiscal benefits from quitting, such as fewer hospital admissions attributed to smoking, were not incorporated into the model. Fourth, cross-price elasticities of demand were not included as it is possible that waterpipe tobacco smokers may substitute products (including moving to other waterpipe tobacco brands within a market share or between market shares altogether) as a result of rising prices. However, based on data from neighbouring Palestine, Lebanon and Jordan, the cross-price elasticity is likely to be near zero and so we are reasonably assured that the TETSiM model is suitable for an analysis of Egypt’s tobacco market.^32^ Furthermore, raising taxes on all tobacco products simultaneously may discourage product substitution and this is a regular action already undertaken by the Egyptian government, where it increased taxes on all tobacco products and applied taxes on liquids used for electronic smoking devices.^22^ Finally, the model does not consider the impact of raising tax on illicit trade, which is likely to be a concern for policy makers. However, there are several actions that could be undertaken by the government to address this concern.^17^ In this regard, Egypt has already signed the WHO FCTC Protocol to Eliminate Illicit Trade in Tobacco Products in January 2021 (Presidential Decree No. 170/2020).^39^

## Conclusion

Introducing the specific excise tax on waterpipe tobacco in Egypt under either simple (specific excise only) or mixed (specific and ad valorem excise) tax policy approaches can yield considerable government revenue and public health gains, but the simple approach is better in its economic and public health gains than the mixed approach in all proposed tax scenarios, which is in line with the WHO FCTC recommendations. This study provides policy makers with the local and contextualised scientific base for a model that serves different tax scenarios. The scenarios could be framed for political implementation as a phased approach, and the model unfolds potential to improve government financing while protecting public health. To enable more accurate simulations, policy makers are required to provide updated national data on all model parameters. Future models should consider further impacts of waterpipe tobacco taxation on sociodemographic inequalities, and ensure future changes to waterpipe tobacco taxes are subject to regular evaluation.

## Data Availability

All data relevant to the study are included in the article or uploaded as supplementary information.

## References

[1] World Health Organization. WHO report on the global tobacco epidemic 2021: addressing new and emerging product. Available: https://www.who.int/publications/i/item/9789240032095 [Accessed 11 Nov 2021].

[2] WHO Framework Convention on Tobacco Control. Guidelines for implementation of article 6. Available: https://fctc.who.int/publications/m/item/price-and-tax-measures-to-reduce-the-demand-for-tobacco [Accessed 12 Feb 2023].

[3] Marten R, Paul J, Tan Torres Edejer T, et al. Health taxes: a call for papers. BMJ Glob Health 2022;7:e010709. 10.1136/bmjgh-2022-010709 PMC9557789 36192050

[4] World Health Organization. WHO global report on trends in prevalence of tobacco use 2000–2025 4TH Ed. World Health Organization,. 2021 Available: https://apps.who.int/iris/handle/10665/348537 [Accessed 11 Nov 2021].

[5] Global Center for Good Governance in Tobacco Control. Egypt tobacco industry interference index 2021. Available: https://globaltobaccoindex.org/country/EG [Accessed 13 Feb 2023].

[6] Independent arabia. Egypt moves prices of smoke and Moassel next July. an increase every three years for the universal health insurance. 2021. Available: www.independentarabia.com/node/227986/اقتصاد/أخبار-وتقارير-اقتصادية/مصر-تحرك-أسعار-الدخان-والمعسل-في-يوليو-المقبل [Accessed 11 Nov 2021].

[7] World Health organization. Regional office for the Eastern Mediterranean. tobacco free initiative. read our publications. economics. tobacco tax. Egypt. Available: http://www.emro.who.int/tfi/publications/economics.html;%20https://applications.emro.who.int/docs/WHOEMTFI209E-eng.pdf?ua=1 [Accessed 11 Nov 2021].

[8] World Health Organization. Home. Publications overview. 9.3 taxes and retail price for other tobacco products. web annex VI: global tobacco control policy data. 2021. Available: https://www.who.int/publications/i/item/WHO-HEP-HPR-TFI-2021.9.3 [Accessed 11 Nov 2021].

[9] World Health Organization. Egypt global youth tobacco survey 2014. Available: https://extranet.who.int/ncdsmicrodata/index.php/catalog/289 [Accessed 15 Sep 2021].

[10] Egypt National STEPwise Survey for Noncommunicable Diseases Risk Factors Report 2017. A joint report by the Egyptian Ministry of health and population and the world health organization. 2017. Available: https://www.who.int/ncds/surveillance/steps/Egypt_National_STEPwise_Survey_For_Noncommunicable_Diseases_Risk_Factors_2017_Report.pdf?ua=1 [Accessed 18 Sep 2021].

[11] Alwatan News. Addiction treatment and abuse fund: the smoking rate among secondary school students is 12.8. 2022. Available: https://www.elwatannews.com/news/details/5975539 [Accessed 29 Mar 2022].

[12] Shisha and Smokeless tobacco use among university students in Egypt: prevalence, determinants, and economic aspect. A joint report by the Egyptian Ministry of health and population and the world health organization 2014. Available: http://applications.emro.who.int/dsaf/EMROPUB_2014_EN_1752.pdf?ua=1 [Accessed 18 Sep 2021].

[13] Waziry R, Jawad M, Ballout RA, et al. The effects of Waterpipe tobacco smoking on health outcomes: an updated systematic review and meta-analysis. Int J Epidemiol 2017;46:32–43. 10.1093/ije/dyw021 27075769

[14] Eissenberg T. Now is the time for effective regulation regarding tobacco smoking using a Waterpipe (hookah). J Adolesc Health 2019;64:685–6. 10.1016/j.jadohealth.2019.03.011 31122500

[15] Mostafa A. Self-reported addiction to and perceived behavioural control of Waterpipe tobacco smoking and its patterns in Egypt: policy implications. East Mediterr Health J 2020;26:18–28. 10.26719/2020.26.1.18 32043542

[16] WHO Representative Office, Egypt. Dimensions of the problem of tobacco consumption and tobacco control policies oral presentation. civil society and Ngos Allying for tobacco control in Egypt. workshop; 2023 Feb 8.

[17] World Health. WHO technical manual on tobacco tax policy and administration. World Health Organization,. 2021 Available: https://apps.who.int/iris/handle/10665/340659

[18] Jawad M, Awawda S, Abla R, et al. Impact of Waterpipe tobacco taxation on consumption, government revenue and premature deaths averted in Jordan, Lebanon and Palestine: a simulation study. Tob Control 2022:tobaccocontrol-2022-057284. 10.1136/tc-2022-057284 PMC10958304 36601792

[19] Sustainable development knowledge platform. Sustainable development goal 3. progress of goal 3 in 2017 United Nations. Available: https://sustainabledevelopment.un.org/sdg3 [Accessed 19 Sep 2021].

[20] Vision of Egypt. 2030. Available: https://mped.gov.eg/EgyptVision [Accessed 19 Aug 2021].

[21] van Walbeek C. A simulation model to predict the fiscal and public health impact of a change in cigarette excise taxes. Tob Control 2010;19:31–6. 10.1136/tc.2008.028779 19850550

[22] Discussions with the policymakers in the Egyptian tax authority based on the law No.13 of 2020 for amending some provisions of the tax law on the added value promulgated by law No.67 of 2016. Available: https://manshurat.org/node/65981 [Accessed 12 Feb 2023].

[23] World Health. Tobacco use in Shisha: studies on Waterpipe smoking in Egypt. 2006. Available: https://apps.who.int/iris/handle/10665/119837 [Accessed 29 Mar 2022].

[24] Egypt 360. Economics. obliging cigarette companies to write prices. A government plan to increase taxes and confront smuggling. 2021. Available: https://masr360.net/t/إلزام-شركات-السجائر-بكتابة-الأسعار-خط/ [Accessed 8 Feb 2023].

[25] Mostafa A, El Houssinie M, Fotouh AA. Multiple tobacco use among young adult Waterpipe Smokers in Egypt. East Mediterr Health J 2018;24:7–17. 10.26719/2018.24.1.17 29658616

[26] Mostafa A, Mohammed HT, Hussein RS, et al. Do pictorial health warnings on Waterpipe tobacco packs matter? recall effectiveness among Egyptian Waterpipe Smokers & non-Smokers. PLoS One 2018;13:e0208590. 10.1371/journal.pone.0208590 30562376 PMC6298733

[27] Mostafa A, El Houssinie M, Hussein RS. Perceived efficacy of existing Waterpipe tobacco warning labels versus novel enhanced generic and Waterpipe-specific SETS. PLoS One 2021;16:e0255244. 10.1371/journal.pone.0255244 34314460 PMC8315518

[28] Mostafa A, Ismail N. E8-LWDS: factorial structure and Psychometric properties of the Lebanese Waterpipe dependence Scale-11 in 1490 Egyptian Waterpipe tobacco Smokers-A critical approach. Int J Environ Res Public Health 2021;18:6741. 10.3390/ijerph18136741 34201512 PMC8269008

[29] Egypt consumer price index (CPI) Yoy. Available: https://www.investing.com/economic-calendar/egypt-cpi-1198 [Accessed 10 Feb 2023].

[30] Xe Historical Currency Exchange Rates Chart. Egyptian pound to US dollar exchange rate chart. Available: https://www.xe.com/currencycharts/?from=EGP&to=USD [Accessed 10 Feb 2023].

[31] Nassar H, World Bank. The Economics of tobacco in Egypt: A new analysis of demand HNP discussion paper. 2003. Available: http://hdl.handle.net/10986/13716;%20https://openknowledge.worldbank.org/bitstream/handle/10986/13716/288920Nassar010The0Economics010whole.pdf?sequence=1&isAllowed=y [Accessed 11 Nov 2021].

[32] Chalak A, Nakkash R, Abu-Rmeileh NME, et al. Own-price and cross-price Elasticities of demand for cigarettes and Waterpipe tobacco in three Eastern Mediterranean countries: a volumetric choice experiment. Tob Control 2023;32:86–92. 10.1136/tobaccocontrol-2021-056616 34193608 PMC9763177

[33] United Nations. UN Comtrade database. Available: https://comtrade.un.org/data [Accessed 10 Feb 2023].

[34] Doll R, Peto R, Boreham J, et al. Mortality in relation to smoking: 50 years' observations on male British doctors. BMJ 2004;328:1519. 10.1136/bmj.38142.554479.AE 15213107 PMC437139

[35] Analysis of tobacco industry pricing strategies in 23 European Union countries using commercial pricing data. Tob Control 2019;28:e102–9. 10.1136/tobaccocontrol-2018-054826 31028176

[36] Hanafy K, Saleh ASE, Elmallah M, et al. The Economics of tobacco and tobacco taxation in Egypt; International Union against tuberculosis and lung disease Paris, France. 2010. Available: https://www.tobaccofreekids.org/assets/global/pdfs/en/Egypt_tob_taxes_en_F.pdf [Accessed 15 Aug 2021].

[37] Levy DT, Fouad H, Levy J, et al. Application of the abridged Simsmoke model to four Eastern Mediterranean countries. Tob Control 2016;25:413–21. 10.1136/tobaccocontrol-2015-052334 26080365 PMC4681690

[38] Central Agency for Public Mobilization and Statistics. Household income, consumption, and expenditure survey. Available: https://www.capmas.gov.eg/Admin/Pages%20Files/2019123101612income1.pdf [Accessed 5 May 2022].

[39] State Information Services. Your gateway to Egypt home. Presidency. Presidential decrees. Available: https://sis.gov.eg/Story/154025/Presidential-decree-to-eliminate-illicit-trade-in-tobacco-products?lang=en-us [Accessed 20 Aug 2021].

[40] The World Bank. Data. Egypt, Arab Rep. Available: https://data.worldbank.org/country/EG [Accessed 29 Mar 2022].

