# Supplementary material for: Health and economic impacts of introducing specific excise tax to waterpipe tobacco in Egypt: a simulation model of simple and mixed tax policy approaches
Source: BMJ Glob Health. 2023 Oct 9;8(Suppl 8):e012048. doi: 10.1136/bmjgh-2023-012048 (PMC10565201; doi:10.1136/bmjgh-2023-012048)
Supplement: Supplementary data [file bmjgh-2023-012048supp001.pdf]

Supplementary Tables

Table S1. Estimates for Egypt's waterpipe tobacco price elasticity for each of the four market shares

	Lebanon			Jordan			Palestine			Average % difference ([C]+[F]+[I])/3 [J]	Egypt cigarette elasticity [K]	**New Egypt waterpipe elasticity ([K]*(1+([J]/100))**
Market share	PED waterpipe tobacco [A]	PED cigarettes [B]	% difference ([B]-[A])/[B] [C]	PED waterpipe tobacco [D]	PED cigarettes [E]	% difference ([E]-[D])/[E] [F]	PED waterpipe tobacco [G]	PED cigarettes [H]	% difference ([H]-[G])/[H] [I]			
Premium waterpipe tobacco café	-2.312	-1.157[1]	0.998	-0.674	-1.080[1]	-0.376	-1.120	-1.042[1]	0.075	23.2	-0.397	**-0.489**
Premium waterpipe tobacco home	-1.949	-1.157[1]	0.685	-0.601	-1.080[1]	-0.444	-1.209	-1.042[1]	0.812	-19.0	-0.397	**-0.321**
Discount waterpipe tobacco café	-1.699	-0.639[2]	1.659	-0.335	-0.719[2]	-0.534	-0.291	-1.209[2]	-0.759	12.2	-0.397	**-0.445**
Waterpipe tobacco home	-1.700	-0.639[2]	0.166	-0.915	-0.719[2]	0.273	-0.650	-1.209[2]	-0.462	49.0	-0.397	**-0.592**
Reference	[32]										[31]	

Table S2. Change in waterpipe tobacco market share quantity consumed under base case and 75% scenario in Egypt

Market share category	Market share			Number of 20g waterpipe tobacco units		
	Base scenario (%)	75% scenario (%)	Difference (%)	Base scenario (%)	75% scenario (%)	Difference (%)
**Approach 1: Simple specific excise tax structure**						
Café/unflavoured	27.7	29.0	1.3	1,016,622,529	352,197,243	65
Café/flavoured	18.9	44.6	25.7	693,652,195	541,043,160	22
Home/unflavoured	51.3	21.0	-30.3	1,882,770,244	255,266,917	86
Home/flavoured	2.1	5.4	3.3	77,072,466	65,555,694	15
**Approach 2: Mixed specific and *ad valorem* excise tax structure**						
Café/unflavoured	27.7	30.3	2.6	1,016,622,529	384,657,919	62.2
Café/flavoured	18.9	41.4	22.5	693,652,195	526,462,847	24.1
Home/unflavoured	51.3	23.2	-28.1	1,882,770,244	295,155,473	84.3
Home/flavoured	2.1	5.1	3.0	77,072,466	64,397,249	16.4

Table S3. Waterpipe tobacco taxation model sensitivity analyses inputs and impact of different parameter changes on consumption, premature deaths averted, and government revenue, expressed as a percentage relative to the 75% scenario in Egypt

	**Inputs**	**Waterpipe tobacco consumption (%)**	**Premature deaths averted (%)**	**Government tax revenue (%)**
Café unflavored own-price elasticity of demand, lower 95% CI	-0.600	-9.0%	5.7%	-8.8%
Café unflavored own-price elasticity of demand, upper 95% CI	-0.070	41.9%	-26.5%	41.4%
Café flavored own-price elasticity of demand, lower 95% CI	-0.980	-9.8%	9.1%	-10.0%
Café flavored own-price elasticity of demand, upper 95% CI	-0.370	2.8%	-2.6%	2.8%
Home unflavored own-price elasticity of demand, lower 95% CI	-1.170	-18.0%	6.2%	-17.8%
Home unflavored own-price elasticity of demand, upper 95% CI	-0.660	-4.3%	1.5%	-4.3%
Home flavored own-price elasticity of demand, lower 95% CI	-0.810	-1.2%	9.8%	-1.2%
Home flavored own-price elasticity of demand, upper 95% CI	-0.390	-0.2%	1.5%	-0.2%
Cost, insurance, and freight price or ex-factory price, +50%	50%	-1.4%	1.8%	-0.8%
Cost, insurance, and freight price or ex-factory price, -50%	-50%	1.5%	-2.0%	0.9%
Industry overshift, 10%	10.0%	-0.7%	1.0%	-0.7%
Industry undershift, 10%	-10.0%	0.7%	-1.1%	0.8%

CI: confidence interval
